# Cyclic Stretch Induces Inducible Nitric Oxide Synthase and Soluble Guanylate Cyclase in Pulmonary Artery Smooth Muscle Cells

**DOI:** 10.3390/ijms14024334

**Published:** 2013-02-21

**Authors:** Monica R. Shah, Stephen Wedgwood, Lyubov Czech, Gina A. Kim, Satyan Lakshminrusimha, Paul T. Schumacker, Robin H. Steinhorn, Kathryn N. Farrow

**Affiliations:** 1 Department of Pediatrics, Northwestern University, Chicago, IL 60611, USA; E-Mails: monicarshah80@gmail.com (M.R.S.); l-czech@northwestern.edu (L.C.); ginaakim9@gmail.com (G.A.K.); p-schumacker@northwestern.edu (P.T.S.); 2 Department of Pediatrics, University of California, Davis, CA 95817, USA; E-Mails: swedgwood@ucdavis.edu (S.W.); robin.steinhorn@ucdmc.ucdavis.edu (R.H.S.); 3 Department of Pediatrics and Physiology, University of Buffalo, Buffalo, NY 14222, USA; E-Mail: slakshmi@buffalo.edu

**Keywords:** pulmonary vasculature, cyclic stretch, sGC, reactive oxygen species, iNOS

## Abstract

In the pulmonary vasculature, mechanical forces such as cyclic stretch induce changes in vascular signaling, tone and remodeling. Nitric oxide is a potent regulator of soluble guanylate cyclase (sGC), which drives cGMP production, causing vasorelaxation. Pulmonary artery smooth muscle cells (PASMCs) express inducible nitric oxide synthase (iNOS), and while iNOS expression increases during late gestation, little is known about how cyclic stretch impacts this pathway. In this study, PASMC were subjected to cyclic stretch of 20% amplitude and frequency of 1 Hz for 24 h and compared to control cells maintained under static conditions. Cyclic stretch significantly increased cytosolic oxidative stress as compared to static cells (62.9 ± 5.9% *vs.* 33.3 ± 5.7% maximal oxidation), as measured by the intracellular redox sensor roGFP. Cyclic stretch also increased sGCβ protein expression (2.5 ± 0.9-fold), sGC activity (1.5 ± 0.2-fold) and cGMP levels (1.8 ± 0.2-fold), as well as iNOS mRNA and protein expression (3.0 ± 0.9 and 2.6 ± 0.7-fold, respectively) relative to control cells. An antioxidant, recombinant human superoxide dismutase (rhSOD), significantly decreased stretch-induced cytosolic oxidative stress, but did not block stretch-induced sGC activity. Inhibition of iNOS with 1400 W or an iNOS-specific siRNA inhibited stretch-induced sGC activity by 30% and 68% respectively *vs.* static controls. In conclusion, cyclic stretch increases sGC expression and activity in an iNOS-dependent manner in PASMC from fetal lambs. The mechanism that produces iNOS and sGC upregulation is not yet known, but we speculate these effects represent an early compensatory mechanism to counteract the effects of stretch-induced oxidative stress. A better understanding of the interplay between these two distinct pathways could provide key insights into future avenues to treat infants with pulmonary hypertension.

## 1. Introduction

At the time of transition from the fetal to neonatal pattern of circulation, blood vessels in the neonatal pulmonary vasculature are acutely exposed to mechanical forces such as shear stress from the movement of circulating blood, intra-luminal stretch determined by pressure from heart propulsions, and extra-luminal stretch induced by respiratory cycles. Cyclic stretch (CS) alters intracellular signaling, leading to changes in vascular tone, often by the production of vasoactive molecules. Physiologic levels of cyclic stretch can also promote vascular remodeling by inhibiting apoptosis in the vascular endothelium and causing mitotic arrest of vascular smooth muscle cells. Recent reports suggest that pathologic levels of stretch increase production of reactive oxygen species (ROS) *via* increased NADPH oxidase activity. These ROS may in turn trigger vasoconstriction in chronic hypertension, stimulate vascular remodeling or angiogenesis, or induce vascular barrier dysfunction in the pulmonary circulation damaged by ventilator-induced lung injury [1,2].

Nitric oxide (NO), a well-studied vasodilator molecule in the pulmonary vasculature, is produced by nitric oxide synthase (NOS) by conversion of L-arginine to L-citrulline, and activates soluble guanylate cyclase (sGC) to increase levels of cyclic guanine monophosphate (cGMP), ultimately causing vasorelaxation. Three distinct isoforms of NOS have been identified: neuronal NOS (nNOS, NOS I), inducible NOS (iNOS, NOS II) and endothelial NOS (eNOS, NOS III). Past studies presumed eNOS to be the predominant source of NO production in the pulmonary vasculature; however, more recent studies suggest that iNOS is an important isoform in generating the NO-mediated fall in pulmonary vascular resistance during the transition of pulmonary circulation from fetal to neonatal life [3]. The iNOS isoform has been shown to be expressed in the airway epithelium and vascular smooth muscle in late-gestation ovine fetal lung, and some groups speculate that cyclic stretch and increased oxygen exposure of distal airway epithelium may directly activate iNOS, leading to vasorelaxation [3,4].

Recent studies from a lamb model of persistent pulmonary hypertension of the newborn (PPHN) suggest that pulmonary vascular sGC activity is diminished [5–7]. Furthermore, PPHN lambs exhibit increased ROS and NADPH oxidase expression [8–11], which are thought to lead to vasoconstriction [9,11,12]. Given these findings, we were surprised to find that PPHN lambs also have markedly increased iNOS protein expression [13]. In the adult systemic vasculature, ROS have also been shown to decrease the expression and activity of sGCβ, the heme-binding subunit of sGC [12]. Thus, we hypothesized that pathologic cyclic stretch as seen in PPHN leads to increased levels of pulmonary vascular ROS, which would in turn decrease sGC expression and activity. Similar to our findings in intact lambs, we now show that in isolated fetal pulmonary artery smooth muscle cells (PASMC), cyclic stretch induces both cytosolic ROS and iNOS expression. However, in contrast to our hypothesis, we found that stretch increased sGCβ expression and activity in PASMC. Furthermore, treatment with recombinant human superoxide dismutase (rhSOD) did not block stretch-induced sGC activity. Instead, inhibition of iNOS with either a potent and selective inhibitor (1400 W) or iNOS siRNA was sufficient to completely block stretch-induced sGC activity, suggesting stretch-induced changes in iNOS activity are responsible for the changes in sGC activity and that these changes are independent of stretch-induced ROS.

## 2. Results

### 2.1. Cytosolic Oxidant Stress Is Increased in PASMC Exposed to Cyclic Stretch

**Figure 1:**
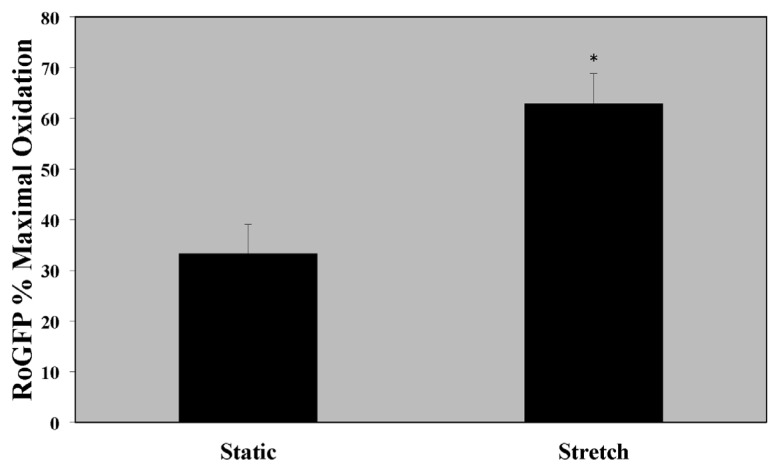
Pulmonary artery smooth muscle cells (PASMCs) exposed to 24 h cyclic stretch have increased oxidative stress in the cytosol. Ovine PASMC were isolated from healthy lambs and exposed to cyclic stretch *in vitro*. Oxidant stress was measured as the % maximal oxidation of the RoGFP probe. Data are shown as mean ± SEM (*n* = 9 for static, *n* = 6 for stretch). * *p* < 0.05.

Previous studies have suggested that fetal and neonatal lambs with chronic intrauterine pulmonary hypertension exhibit increased ROS in their pulmonary arteries [8,13]. Thus, we sought to determine if an *in vitro* model of cyclic stretch would directly increase oxidative stress in PASMC. PASMC exposed to cyclic stretch for 24 h demonstrated a significant increase in cytosolic oxidant stress as measured by the redox-sensitive probe roGFP (Figure 1; stretch *vs.* static: 62.9% ± 5.9% *vs.* 33.3% ± 5.7% oxidized).

### 2.2. rhSOD Does not Block Stretch-Induced Increase in sGC Activity

**Figure 2:**
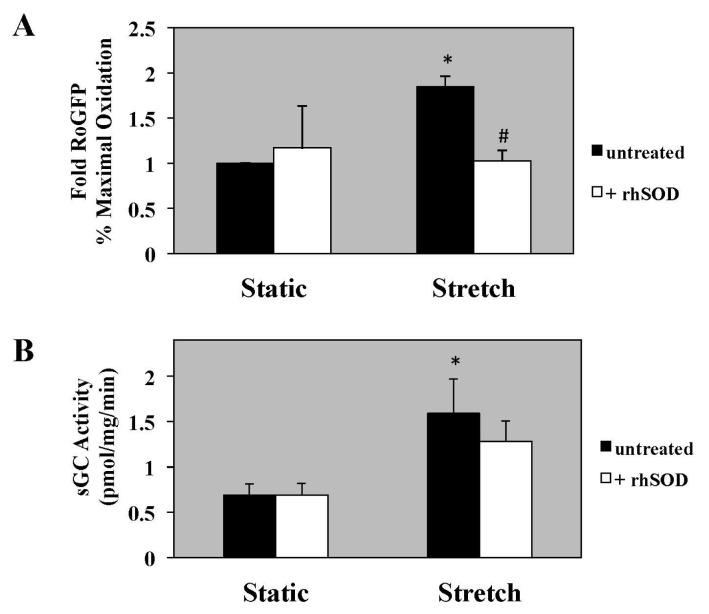
Recombinant human superoxide dismutase (rhSOD) abrogates stretch-induced cytosolic oxidative stress but not soluble guanylate cyclase (sGC) activity in PASMC. Ovine PASMC were isolated from healthy lambs and exposed to cyclic stretch *in vitro* for 24 h. (**A**) Oxidant stress measured in the untreated cells is shown with the black bars, and the cells treated with rhSOD (20 units/mL) are shown with the white bars. Oxidant stress was measured as the % maximal oxidation of the RoGFP probe. Data are shown as mean ± SEM-fold relative to static untreated cells (n = 3). * *p* < 0.05 *vs.* untreated static, ^#^*p* < 0.05 *vs*. untreated stretch. (**B**) sGC activity measured in the untreated cells is shown with the black bars, and the cells treated with rhSOD (20 units/mL) are shown with the white bars. sGC activity was measured as previously described. Data are shown as mean ± SEM (*n* = 9 for static untreated and stretch + rhSOD, *n* = 8 for static+rhSOD and stretch untreated). * *p* < 0.05 *vs.* untreated static.

To determine if the stretch-induced ROS would directly impact sGC activity, we measured both oxidative stress and sGC activity in the presence of the antioxidant, rhSOD. rhSOD blocked stretch-induced cytosolic oxidative stress (Figure 2A; rhSOD-treated stretched PASMC [1.0 ± 0.1-fold] *vs.* untreated stretched PASMC [1.9 ± 0.1-fold]). However, cyclic stretch for 24 h induced sGC activity in untreated PASMC (Figure 2B; 2.3 ± 0.5-fold *vs.* untreated static PASMC), but rhSOD had no impact on the cyclic-stretch induced sGC activity (Figure 2B).

### 2.3. sGC Expression and Activity Is Increased in PASMC Exposed to Cyclic Stretch

**Figure 3:**
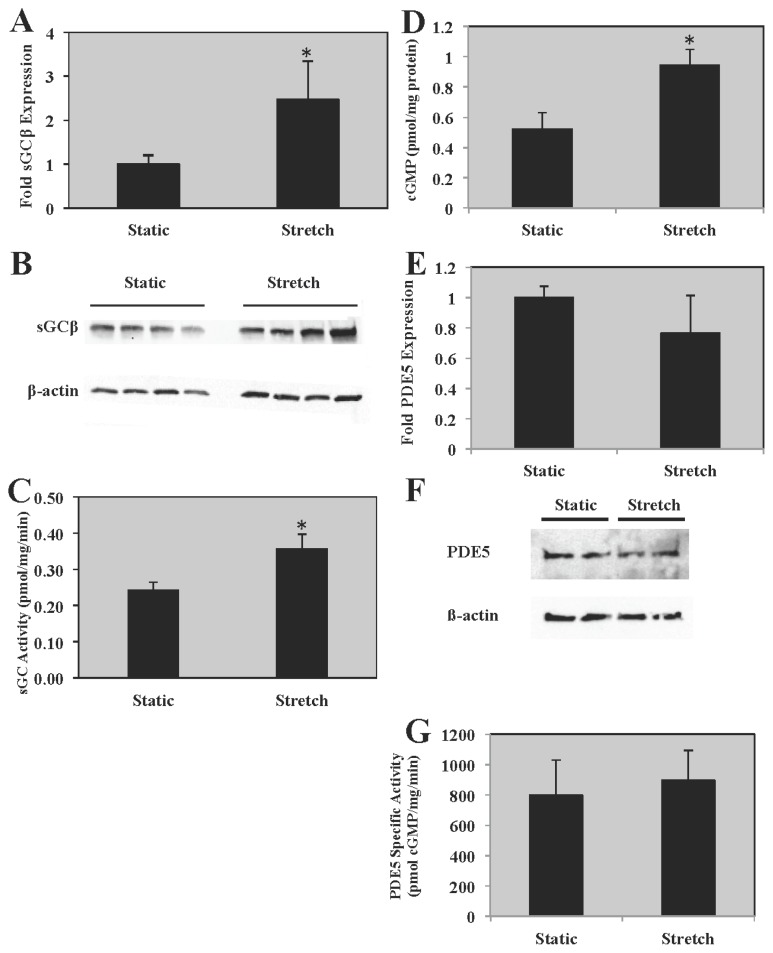
PASMC exposed to 24 h cyclic stretch have increased sGC expression and activity. Ovine PASMC were isolated from healthy lambs and exposed to cyclic stretch *in vitro*. (**A**) sGCβ protein expression was analyzed *via* Western blot, with β-actin normalization. Data are shown as mean ± SEM relative to static cells (*n* = 6). (**B**) Representative Western blot for sGCβ expression. (**C**) sGC activity was measured as previously described, and data are expressed as pmol cGMP produced/mg protein/min. Data are shown as mean ± SEM (*n* = 24). * *p* < 0.05. (**D**) cGMP levels were measured as previously described, and data are expressed as pmol cGMP/mg protein. Data are shown as mean ± SEM (*n* = 6). * *p* < 0.05. (**E**) PDE5 protein expression was analyzed *via* Western blot, with β-actin normalization. Data are shown as mean ± SEM relative to static cells (*n* = 6). (**F**) Representative Western blot for PDE5 expression. (**G**) PDE5 specific activity was measured as previously described, and data are expressed as pmol cGMP hydrolyzed/mg protein/min. Data are shown as mean ± SEM (*n* = 7).

Previous studies in adult systemic SMC and in fetal and lamb resistance pulmonary arteries suggest that elevated ROS are associated with decreased sGC expression and activity [7,12,14]. Thus, we measured sGC expression and activity after exposure to cyclic stretch. PASMC exposed to 24 h of cyclic stretch had increased sGCβ expression (Figure 3A; 2.5 ± 0.9-fold). A representative Western Blot is shown in Figure 3B. Accordingly, we also show that sGC activity increases following exposure to cyclic stretch (Figure 3C; 1.5 ± 0.2-fold). Consistent with increased sGC activity, we note increased cGMP levels in the PASMC exposed to 24 h of cyclic stretch *vs.* static PASMC (Figure 3D; 0.9 ± 0.1 *vs.* 0.5 ± 0.1 pmol cGMP/mg protein). Finally, we demonstrate that 24 h of cyclic stretch does not impact PDE5 protein expression (Figure 3E,F) or PDE5 specific activity (Figure 3G).

### 2.4. iNOS mRNA and Protein Expression Is Increased in PASMC Exposed to Cyclic Stretch

**Figure 4:**
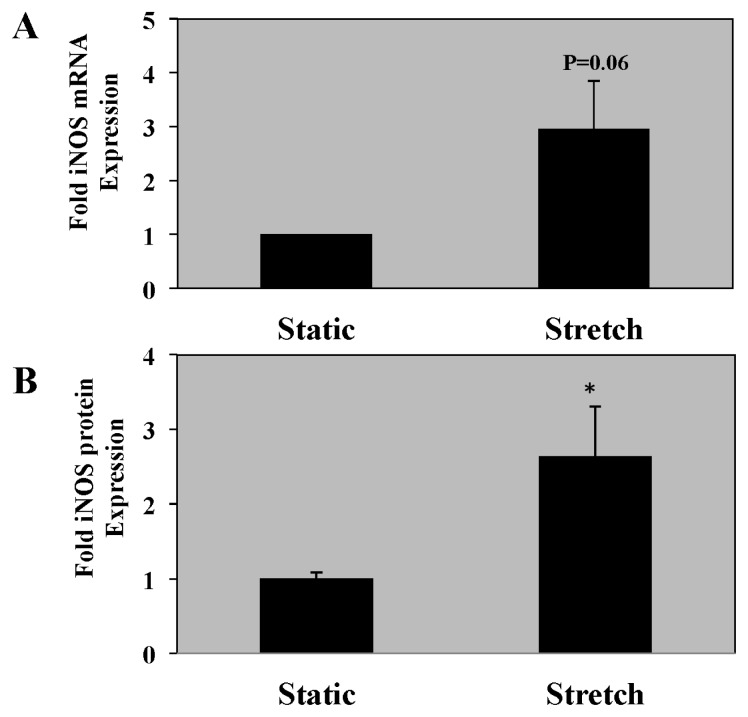
PASMC exposed to 24 h of cyclic stretch have increased inducible nitric oxide synthase (iNOS) mRNA and protein expression. Ovine PASMC were isolated from healthy lambs and exposed to cyclic stretch *in vitro*. (**A**) iNOS mRNA expression is shown with the black bars. iNOS mRNA expression was measured *via* real-time PCR normalized to β-actin. Data are shown as mean ± SEM-fold relative to static cells (*n* = 3). (**B**) iNOS protein expression was analyzed via Western blot, with β-actin normalization (*n* = 14). Data are shown as mean ± SEM relative to static cells. * *p* < 0.05.

We have previously demonstrated that lambs with chronic intrauterine pulmonary hypertension have increased iNOS expression, and others have previously shown mechanical stretch can induce iNOS expression and activity in bronchial or synovial cells [15,16]. Thus, we sought to determine if iNOS activity is directly responsible for the stretch-mediated changes in sGC activity. PASMC exposed to 24 h of cyclic stretch had a trend towards increased iNOS mRNA expression (Figure 4A; 3.0 ± 0.9-fold, *p* = 0.06) and significantly increased iNOS protein expression (Figure 4B; 2.6 ± 0.7-fold, *p* < 0.05).

### 2.5. iNOS Inhibition with 1400 W and iNOS-Specific siRNA Blocks Stretch-Induced sGC Activity

**Figure 5:**
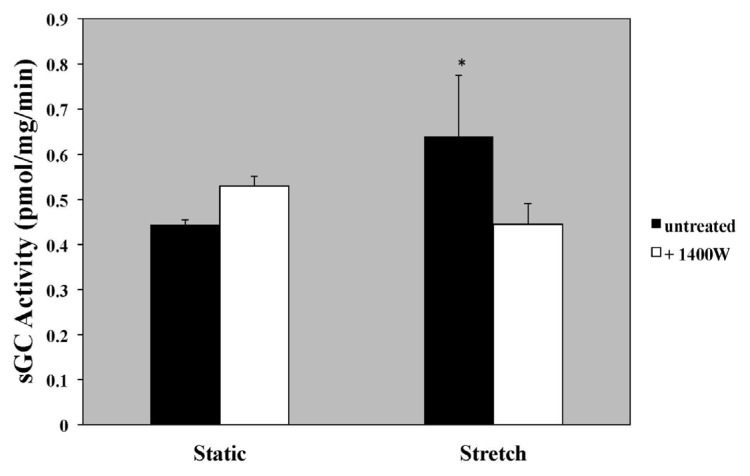
An iNOS inhibitor, 1400 W, decreased sGC activity in PASMC exposed to 24 h of cyclic stretch. Ovine PASMC were isolated from healthy lambs and exposed to cyclic stretch *in vitro*. sGC activity measured in the untreated cells is shown with the black bars and in the cells treated with 1400 W (500 nM) is shown with the white bars. sGC activity was measured as previously described. Data are shown as mean ± SEM relative to untreated, static cells (*n* = 13). * *p* < 0.05 *vs.* untreated static.

**Figure 6:**
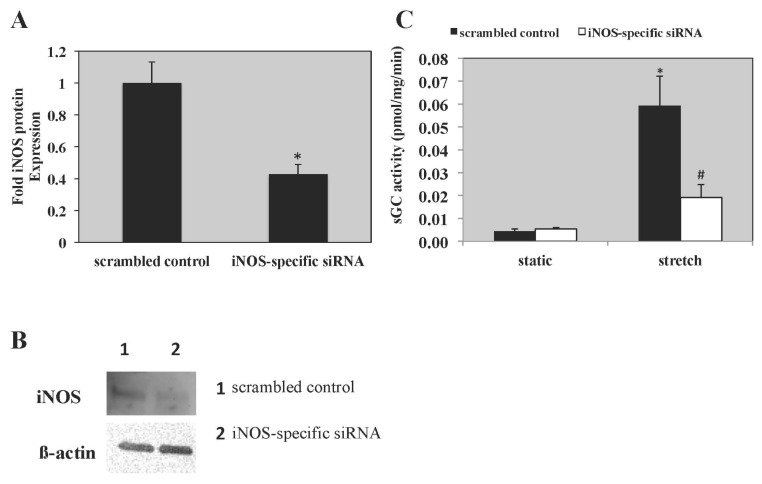
An iNOS-specific siRNA decreased sGC activity in PASMC exposed to 24 h of cyclic stretch. Ovine PASMC were isolated from healthy lambs and exposed to cyclic stretch *in vitro*. (**A**) iNOS protein expression was analyzed *via* Western blot, with β-actin normalization, in static PASMC (*n* = 7). * *p* < 0.05. (**B**) Representative Western blot for iNOS expression in static PASMC treated with an iNOS-specific siRNA. Lane 1 represents scrambled controls and Lane 2 represents PASMC treated with iNOS-specific siRNA. (**C**) sGC activity measured in the scrambled controls is shown with the black bars and in the cells treated with an iNOS-specific siRNA (100 nM) is shown with the white bars. sGC activity was measured as previously described (*n* = 6). * *p* < 0.05 *vs.* untreated static, ^#^*p* < 0.05 *vs*. untreated stretch.

The specific iNOS inhibitor, 1400 W, led to a 30% decrease in sGC activity in PASMC exposed to 24 h of cyclic stretch *vs.* vehicle-treated PASMC exposed to cyclic stretch (Figure 5). Transfection with iNOS-specific siRNA led to a 58% knockdown in iNOS expression (Figure 6A) and led to a 68% decrease in sGC activity in PASMC exposed to 24 h of cyclic stretch *vs.* scrambled control (Figure 6C). A representative Western Blot for iNOS knockdown in PASMC is shown in Figure 6B.

## 3. Discussion

The exposure of blood vessels to mechanical stress is thought to play an important role in vascular remodeling in both normal physiologic adaptation, such as the neonatal transition to ex-utero life, and in pathological conditions, such as hypertension, atherosclerosis and diabetes [1]. The two main mechanical forces acting on the blood vessel walls are stretch and shear stress from the movement of blood through the vessel lumen. In both the systemic and pulmonary circulation, mechanical stretch exerts its effects through the combined action of pulsatile and tonic components. The tonic component is derived from the baseline contractility of vascular smooth muscle cells, while the pulsatile component is derived from cyclic mechanical strain that is imposed by the heart propulsions. Physiological levels of cyclic stretch are thought to be essential for the maintenance of the vascular smooth muscle cell contractile phenotype, the regulation of vascular tone, and the expression of native constituents of the vascular wall extracellular matrix [1].

Vascular stretch has also been shown to stimulate the generation of ROS by activation of redox-sensitive signaling pathways [1]. Superoxide appears to be the initial species generated in these cell types. The potential sources associated with the increased superoxide production include the family of NADPH oxidases, the mitochondrial electron transport chain, and the xanthine oxidase system. In addition, ROS production by the “uncoupling” of NOS activity has been implicated in the pathogenesis of pulmonary hypertension [1,17]. However, it is not clear which of these potential sources are activated directly by cyclic stretch.

The NO-sGC-cGMP signaling pathway has been implicated in many physiological processes including cell growth and proliferation and vascular homeostasis. sGC contains a prosthetic ferrous heme group that binds NO with very high affinity. When NO binds to the enzyme’s heme moiety, a conformational change occurs, leading to a 200-fold increase in the enzyme’s production of cGMP, which then acts as a second messenger to mediate vascular relaxation through the activation of a cGMP-specific protein kinase [18]. At physiological levels of NO, superoxide has been shown to attenuate the actions of NO by reacting with it to generate peroxynitrite in amounts that are likely to have multiple biological effects such as oxidizing the heme moiety of sGC, thereby rendering it unresponsive to NO-stimulation [18].

Thus, based on the ample evidence for increased ROS and decreased sGC activity in intact lambs with PPHN [7,8], we hypothesized that mechanical stretch applied to an *in vitro* cell culture model would increase ROS, and would decrease sGC expression and activity in our PASMC. Surprisingly, we instead demonstrated that 24 h of exposure to cyclic stretch increased both the expression and activity of sGC. These findings represent the first report of stretch-mediated upregulation of sGC expression and activity. Consistent with this upregulation of sGC activity, we see increased cGMP levels. Interestingly, we saw no change in PDE5 expression or activity in the PASMC exposed to cyclic stretch. This suggests that the stretch-mediated changes in sGC are primarily responsible for the observed changes in cGMP. One key difference between our cell studies and the intact lamb studies was the duration of exposure. We exposed our cells to 24 h of cyclic stretch whereas the lambs had their ductus arteriosus ligated 9–14 days prior to delivery, leading to a more prolonged exposure to the mechanical strain associated with pulmonary hypertension. Additionally, in the intact lamb, there are endothelial cells present, which may or may not respond similarly to isolated PASMC. Thus, we speculate that the increase in sGC expression and activity we observed in this study could represent an early compensatory response in the smooth muscle to increased ROS. Over the prolonged time of mechanical strain in the PPHN lambs, increased iNOS and superoxide generate peroxynitrite, which can subsequently inactivate sGC. Hence, with a prolonged exposure to mechanical stretch, this compensatory response is likely lost due to ROS-mediated targeting of sGC for degradation [12].

**Figure 7:**
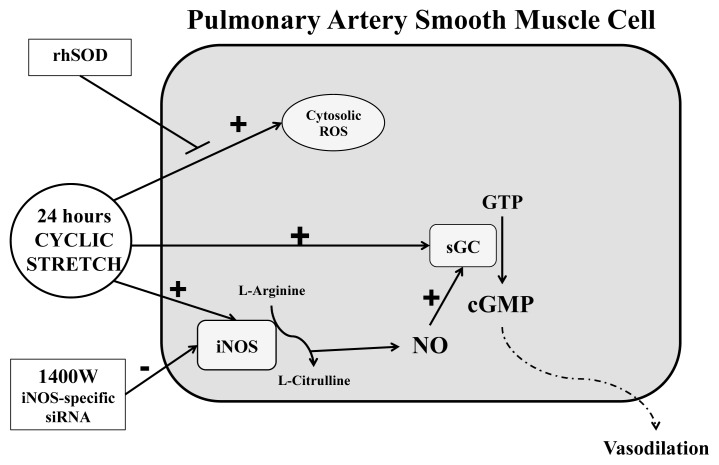
Proposed model for effects of cyclic stretch on ROS and sGC in PASMC. Cyclic stretch increases both cytosolic ROS and sGC expression and activity. The stretch-induced increase in ROS is blocked by rhSOD. The stretch-induced increase in sGC activity is attenuated by 1400 W, a selective iNOS inhibitor, and iNOS-specific siRNA but not rhSOD, suggesting that the stretch-induced changes in sGC are dependent on iNOS and independent of cytosolic ROS.

Since stretch-induced sGC activity was not dependent on ROS in our PASMC, we sought to determine what might be upstream of sGC. sGC expression and activity is well-known to be regulated by NO derived from eNOS [19]. However, our isolated PASMC do not express eNOS, but they do express iNOS and nNOS [13]. Rairigh *et al.* have suggested that iNOS may be an important contributor to the basal production of NO and modulation of basal vascular tone in the normal late-gestation fetal circulation [20]. iNOS is distinct from other isoforms of NOS as its activity is independent of intracellular calcium. Additionally, iNOS is regulated at a transcriptional level primarily through induction by endotoxin and cytokines and is associated with inflammatory states [20]. Interestingly, we have previously demonstrated that PPHN lambs exhibit increased iNOS expression [13]. Others have also demonstrated that cyclic stretch can increase iNOS expression and activity in multiple other cell types [15,16,21]. We now report that in fetal lamb PASMC exposed to 24 h of cyclic stretch, iNOS mRNA and protein expression are up-regulated. We also demonstrate that inhibition of iNOS using either 1400 W, a potent and selective inhibitor of iNOS, or an iNOS-specific siRNA, attenuates stretch-induced sGC activity. We propose that mechanical strain activates iNOS in PASMC, and that increased NO serves to up-regulate sGC activity (Figure 7). These data are in contrast to previously published data that hypoxia induces sGC expression in an iNOS-independent manner in a mouse model of pulmonary hypertension, and highlight that distinct mechanisms may induce pulmonary hypertension under these differing conditions [22].

## 4. Experimental Section

### 4.1. Cell Culture

Primary cultures of ovine PASMC from healthy sheep were isolated and grown to confluence and maintained in DMEM with glucose (Mediatech, Herndon, VA, USA), 10% fetal bovine serum (Hyclone, Logan, UT, USA), antibiotics and antimycotics (Mediatech) at 37 °C in 21% O_2_–5% CO_2_. All experiments were carried out using cells that were less than passage 8, and all experiments were performed with cells isolated from multiple different animals [23]. PASMC were cultured on 6-well BioFlex plates coated with collagen type I (FlexCell, Hillsborough, NC, USA) for 24–48 h. Some cell cultures were treated with recombinant human superoxide dismutase (rhSOD, 20 units/mL diluted in PBS, Sigma, St. Louis, MO, USA), a pharmacologic iNOS inhibitor (1400 W, 500 nM diluted in PBS, Calbiochem, Billerica, MA, USA) or transfected with an iNOS-specific siRNA (100 nM, Invitrogen, Grand Island, NY, USA), and were synchronized prior to the experiment by transfer to serum-free DMEM with antibiotics and antimycotics (Mediatech) prior to the start of cyclic stretch. All untreated cells were treated with the appropriate vehicle (PBS for rhSOD and 1400 W) or transfected with scrambled control siRNA. These cultures were then subjected to biaxial cyclic stretch using the FlexCell 3000 Strain Unit. Plates were placed on a loading station and stretched by applying an oscillatory vacuum to the underside of the membranes with a frequency of 1 Hz and 20% amplitude for 24 h [24]. PASMC were then harvested, and oxidative stress and sGC activity was measured as described below.

### 4.2. Transfection with iNOS-Specific siRNA

PASMC were cultured on Bioflex plates as described above. For transfection assay, 10 μL of an iNOS-specific siRNA (50 μM stock diluted in nuclease-free water, Invitrogen) or scrambled control (50 μM stock diluted in nuclease-free water, Invitrogen) was incubated with 17.5 μL Lipofectamine RNAimax reagent (10 μM stock diluted in nuclease-free water, Invitrogen) in 1.4 mL serum-free DMEM for 20 min at room temperature. Each well of cells received 200 μL of either the iNOS-specific siRNA-transfection mixture or the scrambled control and 500 μL serum-free DMEM such that the final concentration of siRNA or scrambled control was 100 nM; cells were incubated with this transfection mixture for 4–6 h. The cells were then incubated with complete DMEM, and 48 h after transfection, cells were changed to serum-free DMEM with antibiotics and antimycotics and subjected to either cyclic stretch or maintained in static conditions at 37 °C in 21% O_2_–5% CO_2_ for 24 h. PASMC were then harvested, and sGC activity and iNOS expression were measured as described below.

### 4.3. Detection of Reactive Oxygen Species (ROS)

PASMC were infected with 100 PFU/cell of a cytosolic roGFP adenoviral construct. RoGFP is a previously characterized ratiometric fluorescent probe sensitive to cellular oxidative stress [23,25]. To create this probe, surface-exposed residues in green fluorescent protein (GFP) were replaced with cysteine residues capable of forming disulfide bonds. Assessment of fluorescence ratios provides a real-time measure of cysteine thiol redox status in live cells. Ro-GFP infected PASMC were exposed to cyclic stretch or static conditions at 37 °C in 21% O_2_–5% CO_2_ for 24 h, and subsequently, their oxidative status was analyzed using a DakoCytomation CyAn multilaser flow cytometer (Northwestern University Flow Cytometry Core Facility) using 400 nm and 485 nm excitation wavelengths, while emission was assessed at 535 nm, as previously described [23]. For each condition, the cysteine thiol redox status was calculated as percent oxidized, by comparison to values obtained for the fully reduced and fully oxidized conditions.

### 4.4. Soluble Guanylate Cyclase Activity Assay

PASMC were harvested using 1× Mg-lysis buffer (Upstate, Charlottesville, VA, USA) along with protease inhibitor (Sigma) and phosphatase inhibitor (EMD Biosciences, San Diego, CA, USA) as previously described [23]. Cell extracts were sonicated, and protein concentration was determined using the Bradford assay [26]. sGC activity was measured as previously described, and cGMP production was measured as described above in the presence of IBMX, a non-specific phosphodiesterase inhibitor [27]. In brief, 30 μg of total protein was incubated with a previously described incubation buffer containing TrisHCl (0.5 M, pH 7.5, Fisher Scientific, Pittsburgh, PA, USA), MgCl (80 mM, Fisher), IBMX (2 mM, Enzo Life Sciences, Farmingdale, NY, USA), creatinine phosphate (150 mM, Sigma), creatinine phosphokinase (5 mg/mL, Sigma), GTP (10 mM, Sigma), and sodium nitroprusside (10 mM, Sigma) for 10 min at 37 °C. The reaction was terminated by adding ice-cold HCl (0.05 N, Sigma). Precipitate was removed by centrifugation at 2000 × *g* for 15 min. The extracts were dried, and the pellet was re-suspended in EIA buffer (Cayman Chemical, Ann Arbor, MI, USA) as previously described and acetylated according to the manufacturer’s protocol [23]. cGMP content was measured in duplicate, and results were measured using a Labsystems Multiskan EX automated plate reader (Thermo Electron, Pittsburgh, PA, USA) at 420 nm. sGC activity is shown as pmol cGMP produced per mg of total protein per min.

### 4.5. Western Blot Analysis

PASMC were harvested using 1× Mg-lysis buffer, cell extracts were sonicated, and protein concentration determined as described above. Total protein (40 μg) was used to perform Western blots as previously published [23]. Membranes were incubated overnight at 4 °C with primary antibody in 5% milk in 1X TBST at an appropriate dilution (1:500 for rabbit anti-sGCβ [Calbiochem], 1:333 for mouse anti-PDE5 [BD Transduction, San Jose, CA, USA], 1:5000 for mouse β-actin [Sigma] and 1:250 for rabbit anti-iNOS [BD Transduction]). The membranes were washed and incubated for 1–2 h with the appropriate secondary antibody conjugated to horseradish peroxidase diluted to 1:1000. Membranes were washed again and exposed *via* chemiluminesence (Pierce, Rockford, IL, USA). Bands were analyzed using a Digital Science Image Station (Kodak, Rochester, NY, USA). sGCβ or iNOS expression within each western blot was normalized to β-actin. Data are shown as fold relative to untreated static PASMC.

### 4.6. cGMP Enzyme Linked Immunoassay

PASMC steady state cGMP content was measured by enzyme linked immunoassay in duplicate using a commercially available kit (Cayman Chemical) as previously described [23]. Results are shown as pmol cGMP per mg protein for PASMC samples.

### 4.7. PDE5 Activity Assay

PASMCs were harvested, and protein was prepared as described above. Free phosphate was removed from protein extracts by passing extracts over a Centri-Spin 10 column (Princeton Separations, Adelphia, NJ, USA). Protein concentration was determined using the Bradford method. Total protein (5 μg) was assayed for cGMP hydrolytic activity using a commercially available colorimetric cyclic nucleotide phosphodiesterase assay kit (Enzo, Farmingdale, NY, USA) with and without sildenafil (Sigma) as described previously [23]. Results are shown as the PDE5- specific pmol cGMP hydrolyzed per mg total protein per min.

### 4.8. iNOS mRNA Expression

PASMC were harvested for RNA utilizing the Aurum Total RNA Mini Kit (BioRad, Hercules, CA, USA), and RNA was quantified using the Quant-it RiboGreen assay (Molecular Probes/Invitrogen, Carlsbad, CA, USA). cDNA was prepared from total RNA utilizing the iScript cDNA Synthesis Kit (BioRad). Real-time PCR was performed using the iQ SYBR Green Supermix (BioRad) with the iCycler iQ real-time PCR detection system (BioRad) with 38 cycles of real-time data collection using 95 °C for 10 s and 60.5 °C for 45 s, followed by melt curve analysis to verify the presence of a single product. Primers were designed using commercially available software and are based on ovis aries iNOS mRNA accession number AF223942.1: forward 5′-TGAGAATGGCAGCTACTGGGTCAA and reverse 5′-TGGTGATGTCCAGGAAATGGGTGA. Relative iNOS amounts were normalized to β-actin expression using the ΔΔCT method [28].

### 4.9. Statistical Analysis

All data are expressed as the mean ± SEM. Results were analyzed by student’s *t*-test or one-way ANOVA with post-hoc Bonferroni’s analysis as appropriate using Graphpad Prism (San Diego, CA, USA). Statistical significance was set at *p* < 0.05.

## 5. Conclusions

In conclusion, cyclic stretch increases sGC expression and activity in an iNOS-dependent manner in fetal PASMC. The mechanism is not yet known, but does not appear to involve activation by ROS. The increase in sGC expression and activity may represent an early compensatory mechanism to counteract the effects of stretch-induced oxidative stress. This compensation may be lost over time with ongoing cyclic stretch and/or stretch-induced oxidative injury. Therefore, a better understanding of how to preserve sGC activity as well as a better understanding of the interplay between stretch-mediated oxidative injury and the iNOS-sGC pathway in PASMC could provide key insights into future avenues to treat infants with pulmonary hypertension.
